# Integrated analysis of phosphoproteome and ubiquitylome in epididymal sperm of buffalo (*Bubalus bubalis*)

**DOI:** 10.1002/mrd.23432

**Published:** 2020-11-02

**Authors:** Peng‐fei Zhang, Yu‐lin Huang, Qiang Fu, Weng‐tan He, Kai Xiao, Ming Zhang

**Affiliations:** ^1^ State Key Laboratory for Conservation and Utilization of Subtropical Agro‐Bioresources, Animal Reproduction Institute Guangxi University Nanning Guangxi China; ^2^ Department of Cell and Genetics, College of Basic Medicine Guangxi University of Chinese Medicine Nanning Guangxi China

**Keywords:** buffalo epididymis, phosphorylation, proteomics, sperm maturation, ubiquitination

## Abstract

In mammals, sperm need to mature in the epididymis to gain fertilization competency. However, the molecular mechanism underlying buffalo sperm maturation remains elusive. Exploring sperm physiology at the posttranslational modification (PTM) level could help to develop our understanding of these mechanisms. Protein phosphorylation and ubiquitination are major PTMs in the regulation of many biological processes. In the present study, to our knowledge, we report the first phosphoproteome and ubiquitylome of sperm collected from the caput, corpus, and cauda segments of the epididymis using liquid chromatography–mass spectrometry combined with affinity purification. In total, 647 phosphorylation sites in 294 proteins and 1063 ubiquitination sites in 446 proteins were characterized. Some of these proteins were associated with cellular developmental processes and energy metabolic pathways. Interestingly, 84 proteins were both phosphorylated and ubiquitinated, simultaneously. Some of these proteins were involved in, for example, spermatogenesis, reproduction, and spermatid development. Taken together, these data provide a theoretical basis for further functional analysis of phosphorylation and ubiquitination in epididymal sperm of buffalo and other mammals, and serve as an important resource for exploring the physiological mechanism underlying sperm maturation.

## Graphical abstract

Our study investigated the phosphoproteome and ubiquitylome of buffalo epididymal sperm for the first time. Our research develops our understanding of the molecular mechanisms underlying sperm maturation in buffalo.

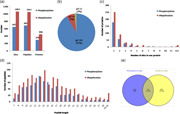

## 1 INTRODUCTION

To acquire progressive motility and the ability of fertilization, spermatozoa released from the testis must transit through a specialized duct called the epididymis (Marchiani et al., 2017). This tissue can be generally separated into three regions based on histological and ultrastructural differences: the caput (head), corpus (body), and cauda (tail; Sullivan et al., 2005). The caput and corpus serve as the sites for early and late sperm maturation, respectively, while the cauda region is responsible for storing functionally mature spermatozoa (Cheng et al., 2018; Dacheux & Dacheux, 2013). During epididymal transit, spermatozoa progressively lose or modify most of their surface proteins and gain new proteins in a well‐organized manner. Numerous high‐throughput proteomic studies have characterized proteins that are possibly involved in the appearance and maintenance of the fertility of the male gamete in the epididymis (Chauvin et al., 2012; Dacheux & Dacheux, 2013; Ijiri et al., 2011; Labas et al., 2015; Sheri et al., 2015). However, the dynamic regulatory events that orchestrate the process of epididymal sperm maturation have yet to be elucidated.

Owing to posttranslational modifications (PTMs), proteins can perform diverse functions. To date, more than 461 distinct PTMs have been described (Khoury et al., 2011), and many proteins have multiple PTMs, and a significant increase in information content would be obtained if PTMs acted in combination (Hunter, 2007). Crosstalk between different types of PTM is an emerging theme in eukaryote research, particularly between phosphorylation and ubiquitination (Hunter, 2007). Phosphorylation, the most widespread and important PTM, regulates many biological processes (Urner & Sakkas, 2003) and is active during epididymal sperm maturation. For example, cAMP‐dependent tyrosine phosphorylation is more active in epididymal sperm in mice (Ecroyd et al., 2004; Lin et al., 2006). The initiation and stimulation of motility for caput epididymal spermatozoa were demonstrated to be induced by the inhibition of Ser/Thr‐protein phosphatase I activity (Silva, 2011; Vijayaraghavan, 1996). Besides, a cSrc family kinase is incorporated into sperm during epididymal transit and is essential for epididymal sperm maturation (Krapf et al., 2012). Protein ubiquitination is another major and conserved PTM, which is known to play a critical regulatory role in many biological processes, such as DNA replication, DNA damage repair, cell cycle, proliferation, and apoptosis (Krapf et al., 2012). The ubiquitin–proteasome system facilitates intracellular protein degradation and serves as the quality control system of the cell (Hochstrasser, 1995). Moreover, ubiquitination might be responsible for eliminating defective spermatozoa during epididymal transit in mammals (Baska et al., 2010; Muratori et al., 2005; Sutovsky et al., 2004, 2001; Vernocchi et al., 2014). The phosphoproteome and ubiquitylome in epididymal sperm maturation have not been studied, to our best knowledge. Thus, comprehensively understanding phosphorylation and ubiquitination through proteome analysis in epididymal sperm is necessary.

Buffalo (*Bubalus bubalis*) is of considerable economic and biological interest especially in southern China; thus, a more robust understanding of the molecular mechanisms underlying epididymal sperm maturation in this ruminant species is of great value. Here, to our knowledge, we report the first phosphorylation and ubiquitination proteomic profiles of buffalo epididymal sperm using liquid chromatography–mass spectrometry (LC–MS/MS). Integrative phosphoproteome and ubiquitylome analyses suggested that epididymal sperm maturation may be partially due to the crosstalk between phosphorylation and ubiquitination. The findings further our understanding of the mechanisms underlying epididymal sperm maturation and offer a new perspective for future research into male reproduction.

## 2 RESULTS

### 2.1 Proteomics analyses of phosphorylation and ubiquitination in epididymal sperm

**Figure 1:**
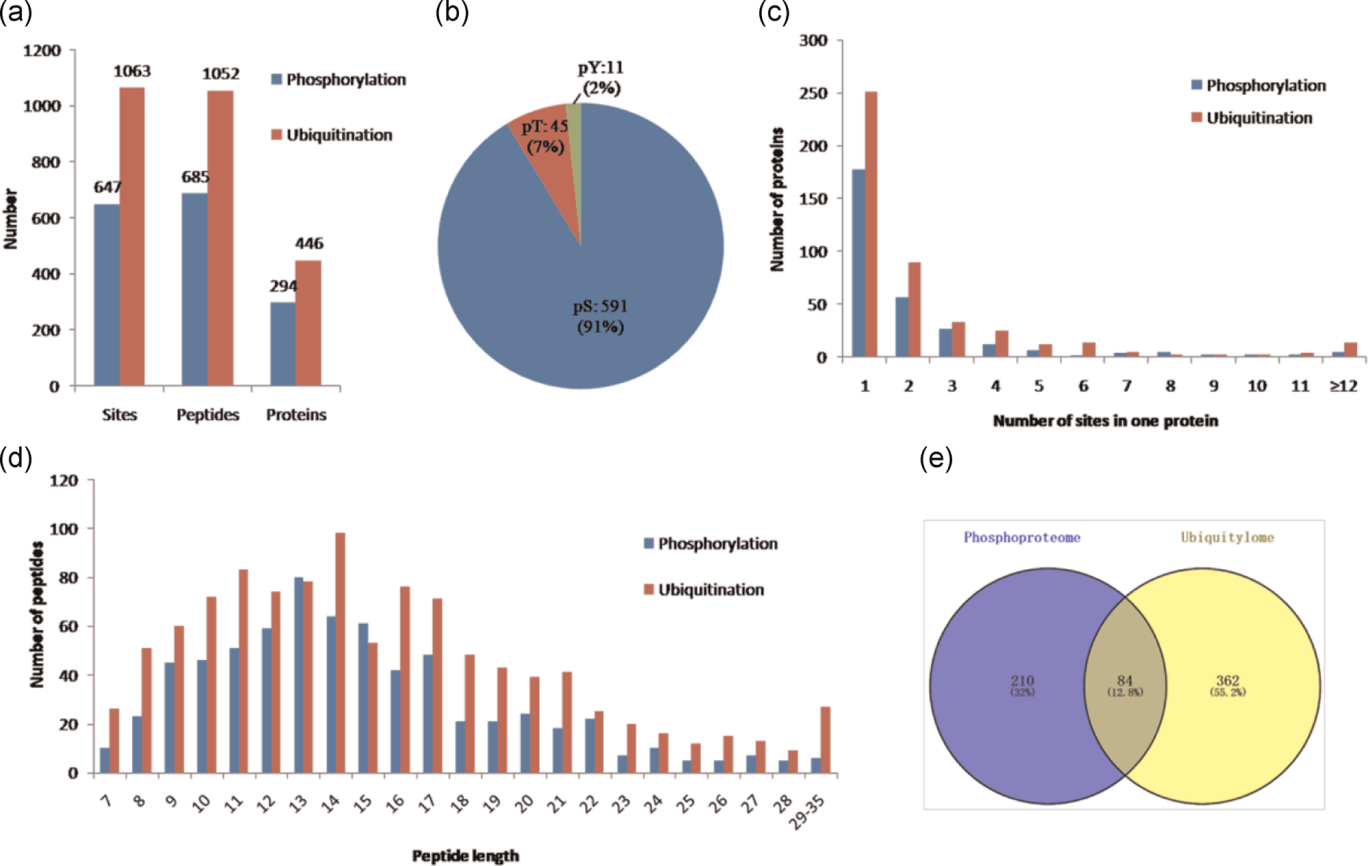
Profile of identified phosphorylated and ubiquitinated sites, peptides, and proteins. (a) The total numbers of phosphorylated and ubiquitinated sites, peptides, and proteins. (b) Distribution of the phosphorylated serine, threonine, and tyrosine residues among the identified phosphorylation sites. (c) Distribution of phosphorylated and ubiquitinated sites in one protein. (d) Distribution of phosphorylated and ubiquitinated peptides based on their length. (e) The total numbers of the overlap between the phosphorylated and ubiquitinated proteins

In the present study, we performed global phosphorylation and ubiquitination proteome analysis of buffalo epididymal sperm using tryptic digestion, affinity enrichment, and LC–MS/MS. In total, 647 phosphorylation sites distributed on 685 peptides in 294 proteins and 1063 ubiquitination sites distributed on 1052 peptides in 446 proteins were identified with high confidence (Figure 1a). Of which, the 647 phosphorylation sites were composed of 591 phosphorylated serine (pS), 45 phosphorylated threonine (pT), and 11 phosphorylated tyrosine residues (Figure 1b). Detailed information for all identified phosphorylation and ubiquitination peptides and their matched proteins are presented in Tables S1 and S2.

We sorted the phosphorylated and ubiquitinated proteins according to the number of phosphorylation and ubiquitination sites, respectively, as shown in Figure 1c. Among the 294 phosphorylated proteins, 177 (60.2%) contained one phosphorylation site and 56 (19%) contained two phosphorylation sites. Notably, 24 (8.2%) proteins contained five or more phosphorylation sites, and eight had at least 10 sites (Table S3). Fibrous sheath interacting protein 2 was the most intensely phosphorylated protein bearing 31 different phosphorylation sites. Moreover, among the 446 ubiquitinated proteins, 251 (56%) had only one ubiquitinated site and 89 (19%) had two ubiquitinated sites. Notably, 50 (11.2%) proteins contained five or more ubiquitination sites, and 18 had at least 10 sites (Table S4). Maestro heat like repeat family member 2B was the most intensely ubiquitinated protein bearing 29 different ubiquitination sites. The length of most phosphorylated and ubiquitinated peptides ranged from 7 to 35 amino acids, which is consistent with the property of tryptic peptides (Figure 1d). Interestingly, we compared the phosphorylation proteins with the ubiquitination data set and found that 84 phosphorylated proteins (Figure 1e and Table 1) were simultaneously ubiquitinated.

**Table 1:** The overlap proteins that found both with phosphorylation and ubiquitination in buffalo epididymal sperm

Protein accession	Protein description	Gene name	Phosphorylation sites	Ubiquitination sites
Q0IID8	1‐Acylglycerol‐3‐phosphate *O*‐acyltransferase 5 (lysophosphatidic acid acyltransferase, epsilon)	AGPAT5	S186	K209, K120
Q58D01	26S proteasome non‐ATPase regulatory subunit 4	PSMD4	S266	K122
P62194	26S proteasome regulatory subunit 8	PSMC5	S395	K222
F1MQJ7	3‐Phosphoinositide dependent protein kinase 1	PDPK1	S242	K258
A6QLG5	40S ribosomal protein S9	RPS9	S153, S23	K30
F1MJS8	A‐kinase anchor protein 3	AKAP3	S246, S821, S485, S157, S52, S210, S33, S177, S103, S36, S179, S183, S205, S611, S765, S61, S75, S431, S215, T212	K71, K250, K41, K220, K180, K60, K691, K433, K616
F1MYH5	A‐kinase anchoring protein 4	AKAP4	S434, S121, S194, S246, S333, S123, S618, S125, S153, S647, S811, S588, S217, S423, S527, S483, S198, S583, S21, S488	K74, K486, K804, K502, K523, K590, K645, K614, K808, K367, K821, K31
F1MQJ0	Angiotensin‐converting enzyme	ACE	S1299	K832, K1016, K570
E1BN43	Ankyrin repeat domain 28	ANKRD28	S1011	K525
E1BP31	Ankyrin repeat domain 42	ANKRD42	S509	K509
E1B717	Armadillo repeat containing 10	ARMC10	S43, S45	K205, K111, K122
F1N4A1	Armadillo repeat containing 12	ARMC12	S91, S48	K136
P33097	Aspartate aminotransferase, cytoplasmic	GOT1	S312	K290
A6H758	C11H9ORF9 protein	SPACA9	S107	K99
Q32L61	Calcium binding tyrosine phosphorylation regulated	CABYR	S331, S335, S240, S243, S118, S427, S367	K5, K242
Q28068	Calicin	CCIN	S391, S12, S58, S97	K400
E1BM27	Calmin	CLMN	S930	K545, K157, K58, K176
P00517	cAMP‐dependent protein kinase catalytic subunit alpha	PRKACA	S140, T198	K30, K48, K280, K310
P00514	cAMP‐dependent protein kinase type I‐alpha regulatory subunit	PRKAR1A	S377, S374	K221, K366, K260, K133
Q32KS3	Capping actin protein of muscle Z‐line alpha subunit 3	CAPZA3	S274, Y289	K89
A6QQM1	CCNY protein	CCNY	S19, S272	K247
Q3ZBU2	CDGSH iron–sulfur domain‐containing protein 1	CISD1	S41	K77, K53, K66
F1MKR7	Cilia and flagella associated protein 77	CFAP77	T178	K51
F1N343	Coiled‐coil domain containing 136	CCDC136	S651, S330, S574, S322, S541, S411, S299, S645	K627, K436, K369, K424, K347, K438, K137, K881, K416, K378, K473
F1MEL4	Coilin	COIL	S259	K477, K460
G3N176	Desumoylatingisopeptidase 1	DESI1	S25	K18
Q0III6	DnaJ homolog subfamily B member 6	DNAJB6	S15	K60
E1BLB4	Dynein axonemal heavy chain 17	DNAH17	T174	K1543
E1B9R5	Dynein axonemal heavy chain 8	DNAH8	S136, S1199, S89, S138, S81	K4081, K578, K2401, K2205, K4551, K1675
F1N7B9	Endophilin‐B1	SH3GLB1	S200	K29
G3X6E2	Family with sequence similarity 166 member A	FAM166A	S147, S187	K63, K61
F1MB15	Fibrous sheath interacting protein 2	FSIP2	S4686, S5359, S1340, S3081, S6426, S6169, S6476, S4716, S6723, S6076, S5976, S3099, S6576, S6245, S4414, S4657, S4176, S6095, S1466, S5213, S4379, S3186, S4546, S6226, S4497, S6726, S4382, S908, S4124, S6715, S5322	K4039, K4098, K3257, K3854, K3673, K6502, K4323, K4058, K4671, K683, K4382
A6QLL8	Fructose‐bisphosphate aldolase	ALDOA	S36, S354	K348, K200, K111
Q3ZBY4	Fructose‐bisphosphate aldolase	ALDOC	S45	K111
Q8MJN0	FUN14 domain‐containing protein 2	FUNDC2	S44, S54, S33, S152	K63, K162, K151, K160, K62, K171
F1MUX6	Glutathione *S*‐transferase	GSTM3	S143, S77	K170, K161, K181, K71, K40, K199, K193, K38, K132, K19, K31, K55
Q0VCS8	Glutathione *S*‐transferase, theta 3	GSTT3	S25	K190, K23
P10096	Glyceraldehyde‐3‐phosphate dehydrogenase	GAPDH	S149	K261, K213
Q2KJE5	Glyceraldehyde‐3‐phosphate dehydrogenase, testis‐specific	GAPDHS	S269, S300, S210, T296	K274, K221, K204, K278, K318, K167
Q76LV2	Heat shock protein HSP 90‐alpha	HSP90AA1	S52	K58, K69, K74, K84, K185, K420, K408, K112, K209, K191, K224, K284, K293, K616
E1BNY9	HECT, UBA and WWE domain containing 1, E3 ubiquitin protein ligase	HUWE1	S3761, S3758, S1852	K1733
F1MWF0	Huntingtin interacting protein 1	HIP1	S764	K621, K660, K775, K977, K604, K527, K575, K927, K730, K1033
Q32KR7	Hypothetical LOC539526	SAXO1	S330, S450, T67	K135
A7MBC5	IARS protein	IARS	S827	K1000, K1042
F1MCM7	IQ motif containing N	IQCN	S711, S737, S764, S724, S1096	K888
E1BM42	KIAA1468	RELCH	S49, S52, S176	K873
E1BNS9	l‐Lactate dehydrogenase	LDHC	S321, S161, S105	K232
F1MLS4	Membrane spanning 4‐domains A14	MS4A14	S349, S283	K492, K547, K541
F1N1D6	Metaxin‐1	MTX1	S50	K41
F1MZU2	*N*‐ethylmaleimide sensitive factor, vesicle fusing ATPase	NSF	S437	K728, K161, K529, K266, K293, K469
E1BCV4	Nucleoporin 98	NUP98	S607	K796, K756, K807
E1BHV9	Ornithine decarboxylase antizyme 3	OAZ3	S55, S9, Y11	K17
Q2T9U2	Outer dense fiber protein 2	ODF2	S37, S74, S139, S237, S101, S129, S261, S244, S645, S58, S632, S73, T172, T177, T31, T39	K259, K239, K253, K259, K609
E1BJM5	PAS domain containing serine/threonine kinase	PASK	S158	K1035, K1046, K218, K1131
F1N0B2	Phospholipid‐transporting ATPase	LOC536660	S855	K152, K704, K229
A4FUZ3	Proteasome (prosome, macropain) 26S subunit, ATPase, 1	PSMC1	S244	K178
F1MKX4	Proteasome activator complex subunit 4	PSME4	S293, S294	K918, K636, K56, K36, K1501, K574
Q3ZCK9	Proteasome subunit alpha type‐4	PSMA4	S7	K54
Q58DN4	Protein phosphatase methylesterase 1	PPME1	S42	K328
E1BC58	RAB2B, member RAS oncogene family	RAB2B	S67, S202	K120, K29
F1N058	Ropporin‐1	ROPN1	S106, S56, S62	K6, K73, K139, K23, K199
F1MX05	Saccharopine dehydrogenase‐like oxidoreductase	SCCPDH	S406	K220, K257
Q0VD22	Serine/threonine‐protein kinase 33	STK33	S87	K373
Q3SZY8	Small membrane A‐kinase anchor protein	‐‐‐	S24, S40	K20
Q32L54	Sperm‐associated antigen 6	SPAG6	S490	K457, K438
F1MI43	Sperm surface protein Sp17	SPA17	S54	K52
F1MUB8	Spermatogenesis and centriole‐associated 1	SPATC1	S252, S330, S337, S238, S312	K421, K38, K483, K471, K430
Q3SZQ3	Spermatogenesis‐associated protein 19, mitochondrial	SPATA19	S133, S84, S142, S91, S37, S59, S26, S116, S138, S107, S82	K83
Q3T0K2	T‐complex protein 1 subunit gamma	CCT3	S414	K128, K507
Q3ZCI9	T‐complex protein 1 subunit theta	CCT8	S261, S317	K326, K62
A0JNM2	Thioredoxin	TXNDC8	S51	K72
E1BJD3	Transmembrane protein 190	TMEM190	S127	K131
E1BCX4	Tripartite motif containing 42	TRIM42	S124	K697, K680
P81947	Tubulin alpha‐1B chain	‐‐‐	S48	K370
Q3MHM5	Tubulin beta‐4B chain	TUBB4B	S115, S40	K350, K122, K58, K336, K362, K324, K154, K216, K297
F1ME38	Ubiquitin like modifier activating enzyme 6	UBA6	S697	K503, K729, K537, K978, K796, K628, K644, K739, K709, K368, K1003, K746, K800, K58, K531, K714, K652, K687, K871, K544
A3KMV5	Ubiquitin‐like modifier‐activating enzyme 1	UBA1	S810	K635
F1MHK9	Uncharacterized protein	LOC524391	S823	K1627, K692, K761
F1ML59	Uncharacterized protein	NT5C1B	S75, S53, S129, S68, S131, S43, S202, T151, T195	K225
F1N3R5	Uncharacterized protein	AQP7	S14	K24, K15
F1N6K8	Uncharacterized protein	LOC789612	S17	K253
F6Q0K7	Uncharacterized protein	SPATA32	S337	K206, K258
G3X752	Vesicle‐associated membrane protein 3	VAMP3	S61	K38, K69, K45
P68002	Voltage‐dependent anion‐selective channel protein 2	VDAC2	S115	K120, K285

### 2.2 Functional annotation and pathway analysis of phosphorylated and ubiquitinated proteins

**Figure 2:**
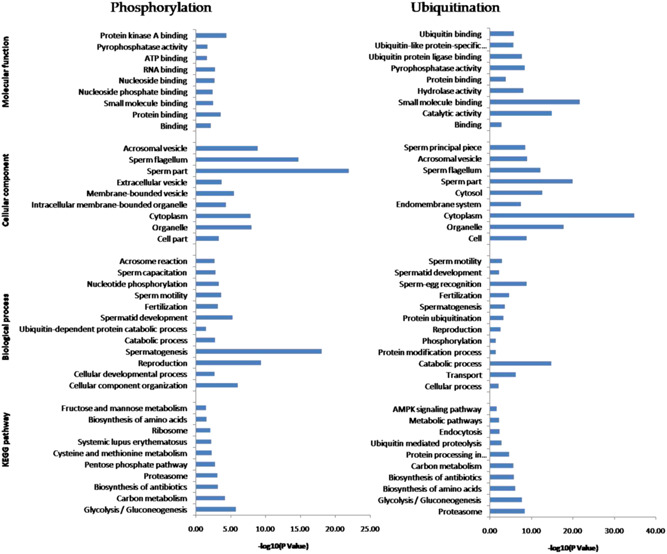
Gene Ontology (GO) annotation and Kyoto Encyclopedia of Genes and Genomes (KEGG) pathway analysis of identified phosphorylated and ubiquitinated proteins. The *x‐axis* represents −log (*p* value), and the *y‐axis* represents the name of GO categories (biology process, subcellular localization, and molecular function) and KEGG pathways

To investigate the possible biological roles of phosphorylated and ubiquitinated proteins, Gene Ontology (GO) and Kyoto Encyclopedia of Genes and Genomes (KEGG) pathway analysis were performed. GO analysis based on the biological process category showed that spermatogenesis, reproduction, and spermatid development were the most significantly enriched in the phosphorylated proteins. However, in the ubiquitinated proteins, the catabolic process was ranked the highest, followed by sperm–egg recognition and transport. Notably, the catabolic process, reproduction, spermatogenesis, spermatid development, fertilization, and sperm motility were enriched in both phosphorylated and ubiquitinated proteins. When enrichment analysis of the cellular components was performed, the phosphorylated proteins were significantly enriched in the sperm part, sperm flagellum, and acrosomal vesicle. These ubiquitinated proteins were enriched significantly on the cytoplasm, sperm part, and organelle. Both phosphorylated and ubiquitinated proteins were significantly enriched in the sperm part. Based on the molecular function category, protein kinase A binding, protein binding, and RNA binding were primarily enriched in the phosphorylated proteins. Nevertheless, the ubiquitinated proteins were mostly enriched in small molecule binding, catalytic activity, and pyrophosphatase activity (Figure 2).

**Figure 3:**
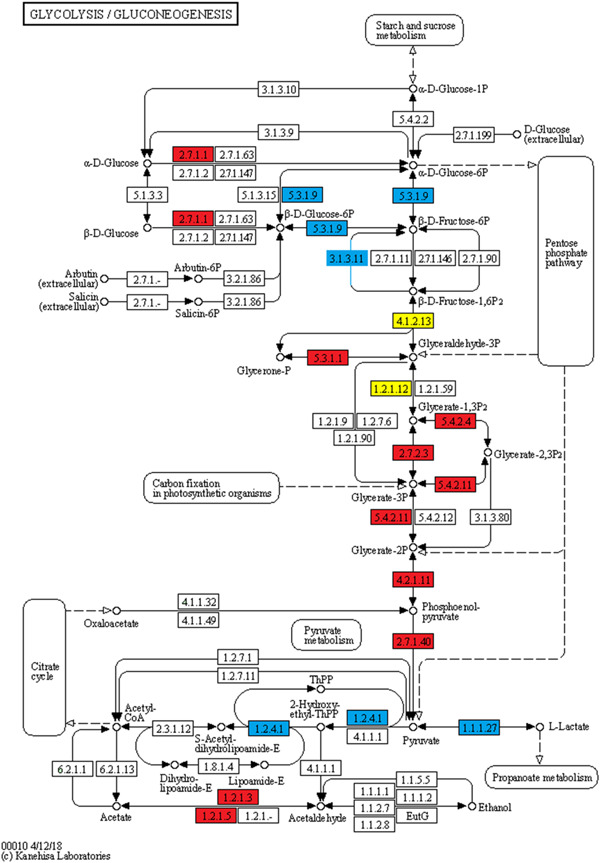
Glycolysis/gluconeogenesis pathway. Blue represents the phosphorylated proteins, red represents the ubiquitinated proteins, and yellow represent proteins both undergo phosphorylation and ubiquitination

**Figure 4:**
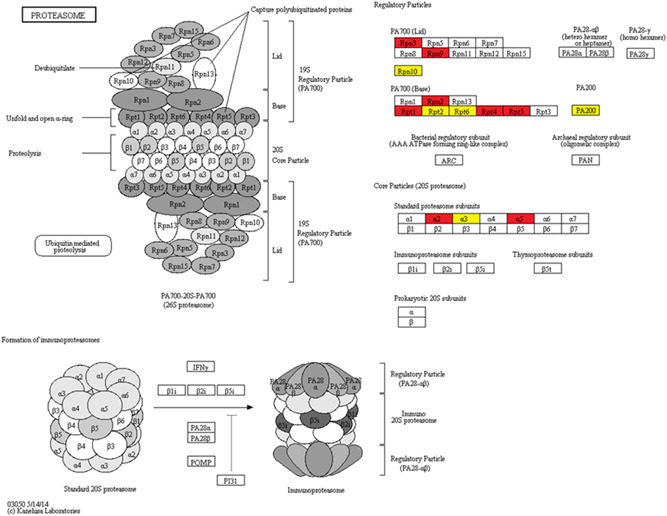
Proteasome pathway. Red represents the ubiquitinated proteins, and yellow represent proteins both undergo phosphorylation and ubiquitination

The KEGG pathway enrichment results showed that glycolysis/gluconeogenesis (Figure 3), proteasome (Figure 4), carbon metabolism, biosynthesis of antibiotics, and biosynthesis of amino acids were significantly enriched in both phosphorylated and ubiquitinated proteins. Additional phosphorylated proteins were mapped to KEGG pathways, including the pentose phosphate pathway, ribosome, and fructose and mannose metabolism. In addition, protein processing in the endoplasmic reticulum, ubiquitin‐mediated proteolysis, and the AMP‐activated protein kinase (AMPK) signaling pathway were also enriched in ubiquitinated proteins (Figure 2).

### 2.3 Integrated analysis between phosphoproteome and ubiquitylome

We compared the phosphoproteome and ubiquitylome data obtained from buffalo epididymal sperm and identified 84 proteins modified with both phosphorylation and ubiquitination (Figure 1e and Table 1). These proteins participated in multiple biological processes, especially spermatogenesis, sexual reproduction, and the catabolic process. The most important cellular components were located in the sperm part, sperm flagellum, and motile cilium. Besides, the significantly enriched molecular functions were protein kinase A binding and small molecule binding. Furthermore, pathways including proteasome, glycolysis/gluconeogenesis, biosynthesis of amino acids, and carbon metabolism were significantly enriched in these 84 proteins (Figure 5a).

Figure 5Integrated analysis between phosphoproteome and ubiquitylome. (a) Gene Ontology annotation and Kyoto Encyclopedia of Genes and Genomes pathway analysis of identified 84 proteins both modified with phosphorylation and ubiquitination. Protein–protein interaction network of phosphorylated and ubiquitinated proteins clustered in (b) proteasome and (c) glycolysis/gluconeogenesis. Blue represents the phosphorylated proteins, red represents the ubiquitinated proteins, and green represents proteins both undergo phosphorylation and ubiquitination. The bubble size represents the degree of interaction

**Figure:**
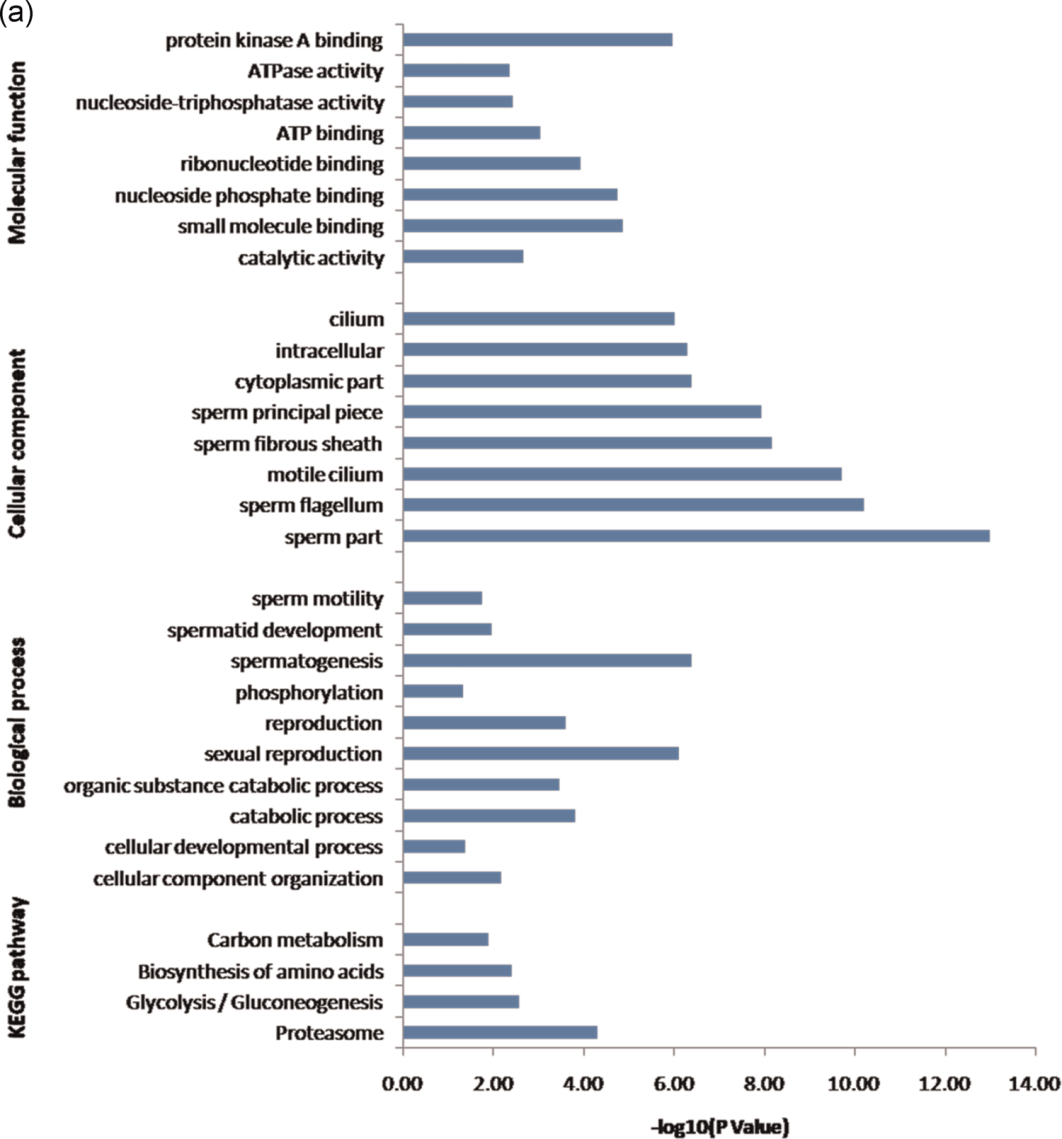

**Figure:**
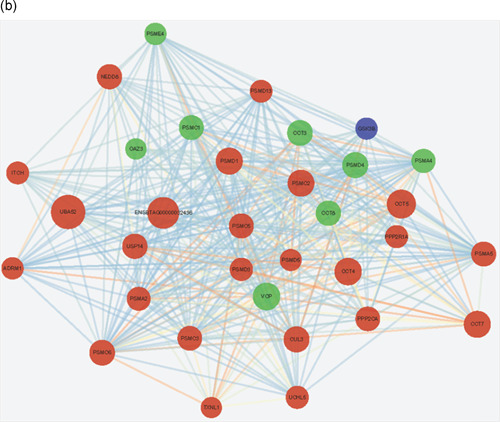

**Figure:**
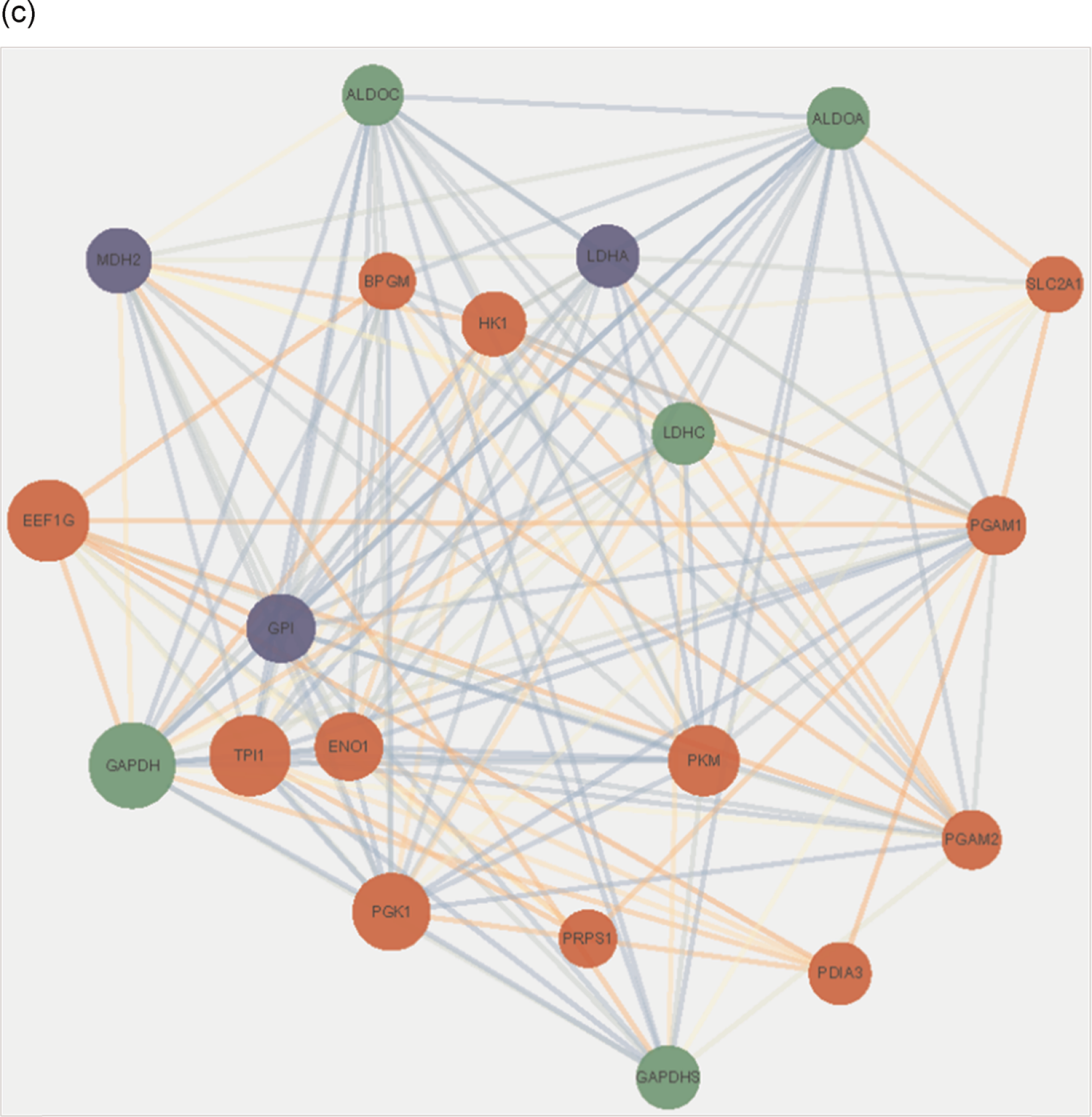

To reveal the crosstalk between phosphoproteome and ubiquitylome in more detail, the protein–protein interaction network based on phosphorylation and ubiquitination proteins was established. The global overview of protein–protein interaction network among phosphorylation and ubiquitination proteins were obtained using the STRING database and Cytoscape software (Figure S1), and two highly interconnected clusters were retrieved (Figure 5b,c). We observed that both phosphorylation and ubiquitination proteins participated in the proteasome and glycolysis pathways. Eight proteins (proteasome 26S subunit ATPase 1 [PSMC1], proteasome subunit alpha type‐4 [PSMA4], 26S proteasome non‐ATPase regulatory subunit 4 [PSMD4], proteasome activator complex subunit 4 [PSME4], T‐complex protein 1 subunit gamma [CCT3], T‐complex protein 1 subunit theta [CCT8], valosin‐containing protein (VCP), and ornithine decarboxylase antizyme 3 [OAZ3]) and five proteins (glyceraldehyde‐3‐phosphate dehydrogenase [GAPDH], glyceraldehyde‐3‐phosphate dehydrogenase‐S [GAPDHS], fructose‐bisphosphate aldolase [ALDOC and ALDOA], and lactate dehydrogenase C [LDHC]) were both phosphorylated and ubiquitylated in proteasome and glycolysis clusters, respectively. These proteins may be of key importance in epididymal sperm and could be selected for further biological investigation.

### 2.4 Confirmation of phosphorylation and ubiquitination of certain proteins

Four candidate total and phosphorylated proteins, PSMA3, PSMA3 (pS250), RAF1, and RAF1 (pS621), were confirmed by immunofluorescence analysis. As shown in Figure 6a, PSMA3 and PSMA3 (pS250) were expressed in the acrosome, neck, and tail of epididymal sperm, RAF1 was mainly expressed in the acrosome and tail, whereas RAF1 (pS621) was only located in the tail. The Western blot results also showed that the proteins PSMA3, PSMA3 (pS250), RAF1, and RAF1 (pS621) were expressed in the buffalo epididymal sperm (Figure 6b). Furthermore, we performed immunoprecipitation (IP) and Western blot analysis to confirm the ubiquitination of two proteins (UCHL1 and HSPA2) from the ubiquitylome data (Figure 6c). The results of the IP/Western blot analyses showed that the proteins UCHL1 and HSPA2 were presented in the ubiquitin‐IP pulldown, and the protein ubiquitin was presented in the UCHL1‐IP and HSPA2‐IP pulldown as well, compared with the immunoglobulin G (IgG) control. These results indicated the reliability of our phosphoproteome and ubiquitylome data set.

Figure 6Verification of the phosphorylation and ubiquitination of certain proteins. (a) Immunofluorescence analysis of proteins PSMA3, PSMA3 (pS250), RAF1, and RAF1 (pS621). PSMA3 and PSMA3 (pS250) are expressed in the acrosome, neck, and tail of epididymal sperm, RAF1 is expressed in the acrosome and tail, while RAF1 (pS621) is only located in the tail of epididymal sperm. Scale bars = 20 μm. (b) Western blot analysis of the proteins PSMA3, PSMA3 (pS250), RAF1, and RAF1 (pS621) in buffalo epididymal sperm. (c) Immunoprecipitation (IP)/western blot analysis of ubiquitinated proteins HSPA2 and UCHL1. Tissue lysates were immunoprecipitated by ubiquitin, HSPA2, and UCHL1 antibodies and the control (immunoglobulin G [IgG]), the ubiquitinated proteins HSPA2 and UCHL1 were presented in the ubiquitin‐IP pulldown and the protein ubiquitin was presented in the UCHL1‐IP and HSPA2‐IP pulldown as well, compared with IgG control by Western blot analysis. DAPI, 4′,6‐diamidino‐2‐phenylindole; FITC, fluorescein isothiocyanate

**Figure:**
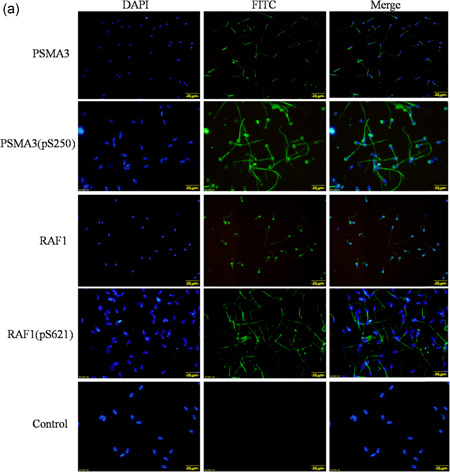

**Figure:**
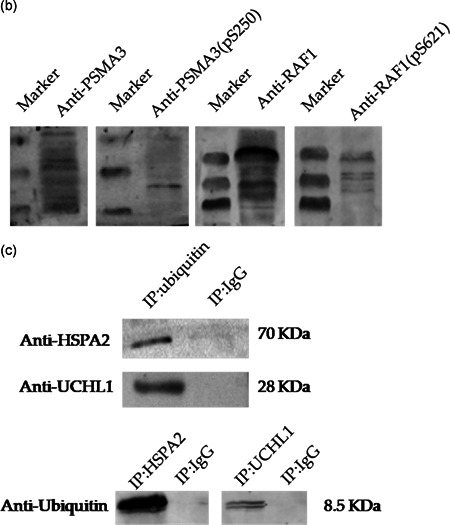

## 3 DISCUSSION

In our study, we successfully identified 647 phosphorylation sites in 294 proteins and 1063 ubiquitination sites in 446 proteins in buffalo epididymal sperm by LC–MS/MS. The phosphorylated and ubiquitinated proteins in epididymal sperm account for 12.5% and 19% of the total proteins (2344, data not shown), respectively. The small number of identified phosphorylated sites may be due to the single‐phosphorylated enrichment method and the nature of epididymal sperm (Kuo et al., 2016). Recently, immunoaffinity reagents that are capable of capturing K‐GG peptides from ubiquitin and its thousands of cellular substrates have been reported (Udeshi et al., 2013; Wagner et al., 2011). A limitation of this approach is that it does not distinguish between various types of modification, such as monoubiquitination, polyubiquitination, and the rare NEDD8 and ISG15 modifications (Bustos et al., 2012; Kim et al., 2011). However, the expression of NEDD8 in mammalian tissues was shown to be developmentally downregulated (Kamitani et al., 1997), and ISG15 expression in bovine tissues was low in the absence of interferon stimulation (Yang et al., 2012). Cell‐culture experiments have shown that most sites identified using di‐glycine‐lysine‐specific antibodies stem from ubiquitylated peptides. Thus, most di‐Gly remnants derived from cellular peptides are derived from ubiquitinated proteins (Kim et al., 2011). Therefore, in the present study, we referred to all di‐Gly modified lysines as “ubiquitination sites,” even though a small portion of these sites may have been derived from a NEDD8 or an ISG15 modification.

Until recently, the phosphoproteome and ubiquitylome were most frequently reported in mammalian cells and tissues in human and murine (Danielsen et al., 2011; Huttlin et al., 2010; Kim et al., 2011; Martin‐Hidalgo et al., 2020; Qi et al., 2014; Wagner et al., 2012; Zahedi et al., 2008). A comparative phosphoproteome was performed to find the phosphorylation difference between buffalo epididymal sperm (Table S1) and mouse (Huttlin et al., 2010; https://phosphomouse.hms.harvard.edu/index.php). It revealed that 164 phosphorylation peptides were common and 483 were newly identified. The common phosphorylation proteins were significantly enriched in sexual reproduction, male gamete generation, cellular component assembly, and DNA packaging. Most of the newly identified phosphorylation proteins in buffalo epididymal sperm were associated with various reproduction processes, such as spermatogenesis, gamete generation, and multicellular organism reproduction (Table S5). Meanwhile, a comparative ubiquitome was performed between buffalo epididymal sperm (Table S2) and mouse (Wagner et al., 2012; Table S6), it revealed that 284 ubiquitination peptides were common and 779 were newly identified. Further analysis demonstrated that the common ubiquitination proteins were significantly enriched in a variety of catabolic processes, while the newly identified ubiquitination proteins were associated with catabolic processes, sperm–egg recognition, sexual reproduction, fertilization, and spermatogenesis (Table S7). This indicated that phosphorylation and ubiquitination are conserved in mammalian and play both common and specific roles in different cells or tissues in different mammalian species.

We annotated phosphorylated and ubiquitinated proteins to specific cellular processes and pathways based on bioinformatics analysis. In the reproduction process, 35 proteins were phosphorylated, 37 were ubiquitinated, and 13 of these were both phosphorylated and ubiquitinated. For example, calicin (CCIN) is found in a diverse range of mammals and is a basic cytoskeletal protein of sperm cells (Paranko, 1990; Paranko et al., 2010). In our study, four phosphorylation sites (S391, S12, S58, and S97) and one ubiquitination site (K400) were found in protein CCIN. Besides, the deficiency in ROPN1 protein in mouse sperm can significantly reduce its fertility (Fiedler et al., 2013). Our previous research demonstrated that ROPN1 was specifically localized in the principal piece of buffalo spermatozoa (Huang et al., 2016). Here, we identified three phosphorylation sites and five ubiquitination sites in ROPN1. Spermatogenesis‐associated protein 19 (SPATA19) is crucial for sperm mitochondrial function and male fertility (Mi et al., 2015). Eleven sites (S133, S84, S142, S91, S37, S59, S26, S116, S138, S107, and S82) were phosphorylated in this protein, while only one site (K83) was ubiquitinated. This may be due to the intense phosphorylation of protein SPATA19 during sperm maturation in buffalo. Sperm surface protein Sp17 (SPA17) was the zona pellucida binding protein on the sperm surface and contributed to the high‐affinity binding between the sperm and zona pellucid (Wen et al., 1999; Yamasaki et al., 1995). In the present study, we verified that SPA17 was ubiquitinated at K52, as well as activating phosphorylation at S54.

In addition, four proteins (CAPZA3, ROPN1, PSME4, and PRKACA) in the spermatid developmental process and two proteins (LDHC and AKAP4) participated in sperm motility were both phosphorylated and ubiquitinated. AKAP4, the most abundant protein in the fibrous sheath of the sperm tail, was mainly expressed in the postmeiotic phase of spermatogenesis. Miki et al. (2002) showed that AKAP4 deficiency will lead to the loss of effective motility in sperm. In particular, epididymal sperm proteins carried out acrosome reactions and sperm capacitation mainly by activating phosphorylation. For example, tripartite motif‐containing 36 (TRIM36), equatorin, nuclear transition protein 2 (TNP2), family with sequence similarity 170 member B (FAM170B), septin 4 (SEPT4), and testis anion transporter 1 (SLC26A8) were only phosphorylated in buffalo epididymal sperm. TRIM36 plays a crucial role in the arrangement of somites during *Xenopus* embryogenesis (Yoshigai et al., 2009). Our results indicated that this protein may participate in acrosome reactions and was phosphorylated at Ser267. Previous research has shown that SEPT4 mutant sperm are defective in the elimination of residual cytoplasm during sperm maturation (Kissel et al., 2005). In our research, buffalo sperm capacitation may relate to the activation of phospho‐site Ser315 of SEPT4.

The glycolysis/gluconeogenesis pathway is a primary source of energy for sperm motility (Nascimento et al., 2008; Tourmente et al., 2015). GAPDH, GAPDHS, ALDOC, ALDOA, and LDHC were simultaneously phosphorylated and ubiquitinated in buffalo epididymal sperm. GAPDH activity was a parameter for determining sperm motility (Fu et al., 2017). GAPDHS, a sole GAPDH isozyme in sperm with four phosphorylated sites and six ubiquitinated sites identified, is required for sperm motility and male fertility (Miki et al., 2004). Two additional glycolytic enzyme subunits, fructose‐bisphosphate aldolase ALDOC and ALDOA, are tightly bound to the fibrous sheath of mouse spermatozoa and essential for sperm motility (Krisfalusi et al., 2006). LDHC is abundant in spermatocytes, spermatids, and sperm and is also required for male fertility (Odet et al., 2008). Three phosphorylated sites (S321, S161, and S105) and one ubiquitinated site (K232) were identified in buffalo epididymal sperm in LDHC. Surprisingly, l‐lactate dehydrogenase a chain (LDHA), an LDHC isozyme, phosphorylated at S105 and S161 rather than ubiquitinated in epididymal sperm in our study. LDHA is mainly responsible for the restoration of sperm function and fertility (Tang et al., 2013). Moreover, our previous work showed that EEF1G was expressed in the nucleus of round spermatids in buffalo (Huang et al., 2016). Here, three ubiquitinated sites, K147, K212, and K404, were newly identified in the EEF1G in epididymal sperm.

The proteasome participates in sperm capacitation, fertilization, and acrosome reaction (Kong et al., 2009; Morales et al., 2003; Sawada et al., 2010). A considerable portion of proteins identified in our phosphoproteome and ubiquitylome participated in the proteasome pathway, of which, eight proteins (PSMC1, PSMA4, PSMD4, PSME4, CCT3, CCT8, VCP, and OAZ3) were both phosphorylated and ubiquitinated. PSME4, a proteasome activator, is required for normal spermatogenesis and male fertility (Khor et al., 2006). A few years ago, PSME4 was identified in the mouse epididymal sperm proteome (Skerget et al., 2015). In our study, we first identified two serine phosphorylation sites (S293 and S294) and six lysine ubiquitination sites (K918, K636, K56, K36, K1501, and K574) on PSME4 that may be critical for sperm maturation. PSMD4, the 19S regulatory complex subunit with one phosphorylation site and one ubiquitination site identified, is involved in the sperm–zona pellucida penetration during fertilization (Yi et al., 2010). Meanwhile, two subunits of T‐complex protein 1 (CCT3 and CCT8) are crucial for spermiogenesis (Counts et al., 2017). OAZ3 is expressed specifically in germline cells and is essential for the formation of a rigid connection between the head and tail during spermatogenesis in mice (Tokuhiro et al., 2009). Here, we identified two serine phosphorylation sites S9 and S55, one tyrosine phosphorylation site Y11, and one ubiquitination site K17 in the OAZ3 protein.

In the present study, we first confirmed that the phosphorylated protein PSMA3 (pS250) was expressed in the acrosome, neck, and tail of epididymal sperm, whereas RAF1 (pS621) was only expressed in the tail. The phosphorylation site S250 of PSMA3has not been previously reported in epididymal sperm. Serine 621 has been previously demonstrated as an important phosphorylation site of RAF1, and it plays a crucial role in catalytic activity and negatively regulates the RAF1 protein (Mischak et al., 1996). Furthermore, the ubiquitination of two proteins (UCHL1 and HSPA2) from ubiquitylome data were confirmed by IP and Western blot analysis. UCHL1 plays a key role in mitotic proliferation and differentiation of spermatogonial stem cells and fertilization (Jungkee et al., 2004; Mtango et al., 2012; Wang et al., 2006). One ubiquitination site, K4, was identified in UCHL1 in epididymal sperm. The molecular chaperone HSPA2 has been verified to play a critical role in meiosis, spermatogenesis, male fertility, and sperm–oocyte binding (Bromfield et al., 2015, 2016; Rogon et al., 2014). Here, we identified 10 ubiquitination sites (K‐127, 57, 139, 160, 129, 188, 78, 109, 72, and 89) in HSPA2. The phosphorylation and ubiquitination of these proteins may play important functions in epididymal sperm.

## 4 MATERIALS AND METHODS

### 4.1 Isolation of epididymal sperm and protein extraction

All procedures involving animal treatment used in the present study were based on the Guiding Principles for animal use as described by the Council for International Organizations of Medical Sciences and approved by the Animal Experimentation Ethics Committee of Guangxi University, Nanning, China. Four adult swamp buffalo (*B. bubalis*) without known disease that could affect their fertility was used and four biological replicates were included in the present study. Epididymal tissues were obtained from freshly killed animals at a local commercial slaughterhouse and transported to the laboratory in sterile isotonic saline within 4 h. The epididymis was then defatted and separated from the testis and vas deferens. Sperm were collected from the caput, corpus, and cauda segments of epididymides by cutting the epididymides and extruding the sperm at 37°C into phosphate‐buffered saline (PBS).

The caput, corpus, and cauda epididymal sperm were pooled and centrifuged at 500*g* for 20 min. The pellets were washed three times with PBS by centrifugation at 500*g* for 20 min at room temperature and resuspended in 1 ml PBS. Spermatozoa were then centrifuged at 400*g* for 30 min on 2 ml of 40% Percoll (Solarbio) in PBS to remove contamination. The spermatozoa pellets were washed again with PBS and resuspended in lysis buffer (8 M urea, 2 mM EDTA, and 1% protease inhibitor cocktail), and then sonicated three times on ice using a high‐intensity ultrasonic processor (Scientz) for protein extraction. The remaining debris was removed by centrifugation at 12,000*g* at 4°C for 10 min. Finally, the supernatant was collected, and the protein concentration was determined with a BCA Kit (Solarbio) according to the manufacturer's instructions.

### 4.2 Trypsin digestion

Trypsin digestion was performed as previously (Wei et al., 2019) described with minor modification. Briefly, the protein solution was reduced with 5 mM dithiothreitol for 30 min at 56°C and alkylated with 11 mM iodoacetamide for 15 min at room temperature in the dark. The protein sample was then diluted by adding 100 mM NH_4_HCO_3_ to a urea concentration of less than 2 M. Finally, trypsin was added to the diluted protein sample at 1:50 trypsin‐to‐protein mass ratio for the first digestion overnight and 1:100 trypsin‐to‐protein mass ratio for the second digestion for 4 h. The peptides thus obtained were quantified by Pierce Quantitative Fluorometric Peptide Assay (Thermo Scientific).

### 4.3 Affinity enrichment of phosphorylated peptides

The phosphopeptide was enriched by TiO_2_ as previously described (Wei et al., 2019). A total of 3 mg of tryptic peptides was desalted using Sep‐Pak Classic C18 Columns (Waters) and phosphopeptide enrichment using a High‐Select TiO_2_ phosphopeptide enrichment Kit (Thermo Scientific) following the recommended protocol. Briefly, desalted lyophilized peptides were dissolved in the binding/equilibration buffer provided with the kit and centrifuged to clarify the dissolved peptides. TiO_2_ spin tips were washed twice with wash buffer and equilibrated once with binding/equilibration buffer before loading peptides. Phosphopeptides were allowed to bind to the TiO_2_ resin followed by sequential washing with binding buffer and wash buffer. Finally, bound phosphopeptides were eluted using elution buffer and lyophilized quickly to avoid dephosphorylation.

### 4.4 Affinity enrichment of ubiquitinated peptides

To enrich ubiquitinated peptides, tryptic peptides dissolved in NETN buffer (100 mM NaCl, 1 mM EDTA, 50 mM Tris‐HCl, 0.5% NP‐40, pH 8.0) were incubated with prewashed anti‐K‐ε‐GG beads (lot number PTM‐1104; PTM Biolabs) at 4°C overnight with gentle shaking. Then, the beads were washed four times with NETN buffer and twice with ddH_2_O. The bound peptides were eluted from the beads with 0.1% trifluoroacetic acid thrice. Finally, the eluted fractions were combined and vacuum dried. For LC–MS/MS analysis, the resulting peptides were desalted with C18 ZipTips (Millipore) according to the manufacturer's instructions.

### 4.5 LC–MS/MS analysis

The enriched phosphorylated and ubiquitinated peptides were dissolved in solvent A (0.1% formic acid), directly loaded onto a reversed‐phase analytical column (15 cm long, 75 μm id). The gradient comprised an increase from 10% to 22% of solvent B (0.1% formic acid in 90% acetonitrile) over 40 min, 22%–35% in 12 min, and climbing to 80% in 4 min then holding at 80% for the last 4 min, all at a constant flow rate of 700 nl/min on an EASY‐nLC 1000 UPLC system. The peptides were subjected to an NSI source followed by tandem mass spectrometry (MS/MS) in Orbitrap Fusion^TM^ (Thermo Scientific) coupled online to the ultra‐performance liquid chromatography. The applied electrospray voltage was 2.0 kV. The *m*/*z* scan range was 350–1550 for a full scan, and intact peptides were detected in the Orbitrap at a resolution of 60,000. A data‐dependent procedure that alternated between one MS scan followed by 20 MS/MS scans with 15.0 s dynamic exclusion. The automatic gain control was set at 5E4.

### 4.6 MS/MS data search

The resulting MS/MS data were processed using the MaxQuant search engine (v.1.5.2.8). Tandem mass spectra were searched against the proteomes *Bos taurus* (24,215 sequences) database concatenated with a reverse decoy database. Trypsin specificity was required and a maximum of four missed cleavages was allowed. The mass tolerance for precursor ions was set as 20 ppm in the first search and 5 ppm in the main search, and the mass tolerance for fragment ions was set as 0.02 Da. Carbamidomethyl on Cys was set as the fixed modification, while oxidation on Met as variable modifications, added to the phosphorylation of Ser, Thr, Tyr residue (phosphorSTY), and Gly–Gly modification for lysines as variable modifications in the phosphoproteome and ubiquitylome analysis, respectively. The false discovery rate was adjusted to less than 1%, and the minimum score for modified peptides was set at greater than 40. The site localization probability was set at greater than 0.75.

### 4.7 Bioinformatic analysis

GO annotation proteome was derived from the UniProt‐GOA database (http://www.ebi.ac.uk/GOA/; Huntley et al., 2014), and the lysine ubiquitination proteins were classified by GO annotation involving three categories: biological processes, molecular functions, and cellular components. The KEGG database (Kanehisa et al., 2000) was used to perform pathway analysis. The functional annotation tool of DAVID bioinformatics resources 6.8 (https://david.ncifcrf.gov; Jiao et al., 2012) was used to identify GO terms and KEGG pathways. The protein–protein interaction network among the surveyed proteins was retrieved from the STRING database (version 10.5) with a confidence score of at least 0.7, and the interaction network was visualized in Cytoscape software (Tay et al., 2017).

### 4.8 Immunofluorescence analysis

The immunofluorescence analysis was performed as previously described (Huang et al., 2016) with minor modification. Briefly, the epididymal spermatozoa were placed on gelatin‐coated slides, air‐dried, and fixed with ice‐cold methanol for 10 min at −20°C. The resulting slides were blocked with 5% goat serum (BOSTER) for 2 h at room temperature and then incubated with primary antibodies against PSMA3 (bs‐9352R; Bioss Biotechnology Inc.), PSMA3 (phospho S250; bs‐9353R; Bioss Biotechnology Inc.), RAF1 (bs‐1703R; Bioss Biotechnology Inc.), and RAF1 (phospho S621; ab157201; Abcam) overnight at 4°C at a dilution of 1:100. After washing three times with PBS, the slides were incubated with secondary antibody labeled with fluorescein isothiocyanate (ab6717; 1:200; Abcam) for 1 h at room temperature, and then coverslipped in Prolong Gold antifade reagent with 4′,6‐diamidino‐2‐phenylindole (Life Technologies) and kept in the dark until photographed using an Olympus IX73 inverted fluorescence microscope (Olympus). For the negative control, the primary antibody was replaced with normal rabbit IgG.

### 4.9 IP and Western blot analysis

To confirm the ubiquitination of proteins utilizing the K‐ε‐GG antibody, we performed IP and Western blot analysis. The lysed cell extracts were immunoprecipitated with anti‐ubiquitin antibody (ab105015; Abcam), anti‐HSPA2 antibody (CSB‐PA010824ESR2HU; CUSABIO BIOTECH CO.), anti‐UCHL1 antibody (ab108986; Abcam), or rabbit IgG (ab205718; Abcam) using protein A+G agarose (Beyotime), after shaking at 4°C for 3 h, the supernatant was carefully removed by centrifugation at 1000*g* for 5 min, and the precipitate was washed five times with PBS buffer (8 mM Na_2_HPO4, 1.5 mM KH_2_PO4, 135 mM NaCl, and 2.7 mM KCl), then 20 μl 1X sodium dodecyl sulfate–polyacrylamide gel electrophoresis (SDS‐PAGE) loading buffer (CWBIO) was added to the precipitate and used for electrophoresis after boiling at 100°C for 5 min. After electrophoresis, the SDS‐PAGE gel was transferred onto a polyvinylidene difluoride membrane by a semidry Western blot analysis system (Bio‐Rad). The membrane was blocked with 5% nonfat milk in a TBST solution for 2 h at room temperature and then incubated overnight at 4°C with primary antibody against UCHL1 (7863‐1004; AbD Serotec), HSPA2 (bs‐18080R; Bioss Biotechnology Inc.), and ubiquitin (bs‐7944R; Bioss Biotechnology Inc.) at a dilution of 1:1000. Membranes washed with TBST buffer three times were incubated with horseradish peroxidase‐conjugated secondary antibodies (CWBIO) in TBST buffer for 1.5 h at room temperature. Bands were visualized with an ECL Detection Kit.

## 5 CONCLUSION

In conclusion, the present study is the first to report on the phosphoproteome and ubiquitylome of epididymal sperm in adult buffalo. All the proteins identified from our large‐scale analysis were involved in numerous biological activities. Protein phosphorylation and ubiquitination could play roles as switches to control some key enzyme activities and assure the proper function of epididymal sperm development and maturation. Thus, we also provided molecular targets for the analysis of sperm maturation at the level of PTMs, but further studies are needed to elucidate the regulatory roles of phosphorylation and ubiquitination in epididymal sperm.

## CONFLICT OF INTERESTS

The authors declare that there are no conflict of interests.

## Supporting information


**Figure S1**. The global overview of protein–protein interaction network among phosphorylation and ubiquitination proteins. Blue represents the phosphorylated proteins, red represents the ubiquitinated proteins, and green represents proteins both undergo phosphorylation and ubiquitination. The bubble size represents the degree of interaction.

Supporting information.

Supporting information.

Supporting information.

Supporting information.

Supporting information.

Supporting information.

Supporting information.
