# Supplementary figures and images for: Integrated analysis of phosphoproteome and ubiquitylome in epididymal sperm of buffalo (Bubalus bubalis)
Source: Mol Reprod Dev. 2020 Nov 2;88(1):15–33. doi: 10.1002/mrd.23432 (PMC7894524; doi:10.1002/mrd.23432)

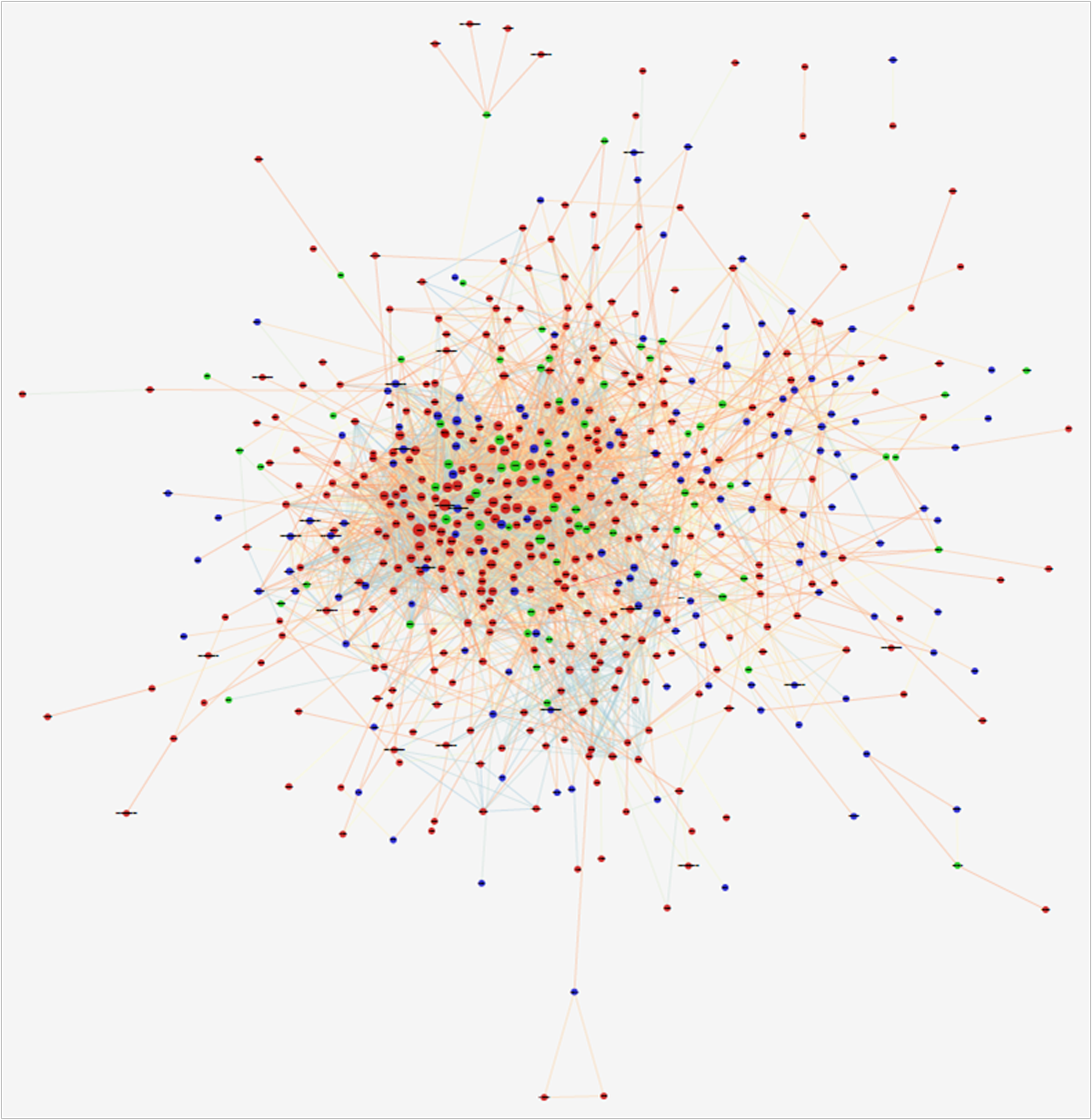

Supplement: Supplementary file 1 — Figure S1. The global overview of protein–protein interaction network among phosphorylation and ubiquitination proteins. Blue represents the phosphorylated proteins, red represents the ubiquitinated proteins, and green represents proteins both undergo phosphorylation and ubiquitination. The bubble size represents the degree of interaction. [file MRD-88-15-s001.tif]
